# Modulatory Effects of Silymarin on Benzo[a]pyrene-Induced Hepatotoxicity

**DOI:** 10.3390/ijms21072369

**Published:** 2020-03-30

**Authors:** Seung-Cheol Jee, Min Kim, Jung-Suk Sung

**Affiliations:** Department of Life Science, Dongguk University-Seoul, Biomedi Campus, 32 Dongguk-ro, Ilsandong-gu, Goyang-si, Gyeonggi-do 10326, Korea; markjee@naver.com (S.-C.J.); pipikimmin@naver.com (M.K.)

**Keywords:** benzo[a]pyrene, BPDE-DNA adduct, silymarin, detoxification, Nrf2, PXR

## Abstract

Benzo[a]pyrene (B[a]P), a polycyclic aromatic hydrocarbon, is a group 1 carcinogen that introduces mutagenic DNA adducts into the genome. In this study, we investigated the molecular mechanisms underlying the involvement of silymarin in the reduction of DNA adduct formation by B[a]P-7,8-dihydrodiol-9,10-epoxide (BPDE), induced by B[a]P. B[a]P exhibited toxicity in HepG2 cells, whereas co-treatment of the cells with B[a]P and silymarin reduced the formation of BPDE-DNA adducts, thereby increasing cell viability. Determination of the level of major B[a]P metabolites in the treated cells showed that BPDE levels were reduced by silymarin. Nuclear factor erythroid 2-related factor 2 (Nrf2) and pregnane X receptor (PXR) were found to be involved in the activation of detoxifying genes against B[a]P-mediated toxicity. Silymarin did not increase the expression of these major transcription factors, but greatly facilitated their nuclear translocation. In this manner, treatment of HepG2 cells with silymarin modulated detoxification enzymes through NRF2 and PXR to eliminate B[a]P metabolites. Knockdown of Nrf2 abolished the preventive effect of silymarin on BPDE-DNA adduct formation, indicating that activation of the Nrf2 pathway plays a key role in preventing B[a]P-induced genotoxicity. Our results suggest that silymarin has anti-genotoxic effects, as it prevents BPDE-DNA adduct formation by modulating the Nrf2 and PXR signaling pathways.

## 1. Introduction

Benzo[a]pyrene (B[a]P) is a ubiquitous environmental pollutant produced during incomplete combustion from sources such as diesel engine exhaust, cigarette smoke, and industrial activities. B[a]P is also produced during certain types of food processing such as grilling and broiling [1]. It is classified by the International Agency for Research on Cancer (IARC) as a group I carcinogen [2,3]. Low-dose B[a]P is constantly absorbed into the body through the inhalation of polluted air, as well as the consumption of charbroiled food. Prolonged exposure to B[a]P accelerates metastasis and angiogenesis in the liver and induces cancer in the liver, lungs, skin, cervix, and the gastrointestinal tract (colorectal and stomach) [4,5,6,7]. Furthermore, polycyclic aromatic hydrocarbons such as B[a]P have been shown to possess very strong bioaccumulation characteristics in animal studies [8,9,10]. After exposure to B[a]P, cellular cytochrome P450 (CYP) metabolizes B[a]P to B[a]P-7,8-dihydrodiol-9,10-epoxide (BPDE), which interacts with DNA to form carcinogenic BPDE-DNA adducts in vitro and in vivo [11,12,13].

In the body, defense systems against xenobiotics such as BPDE include the activation of detoxifying phase II and III enzymes to prevent further cellular damage; specifically, glutathione S-transferases (GSTs) [14], nicotinamide adenine dinucleotide phosphate (NAD(P)H): quinone oxidoreductase 1 (NQO1), sulfotransferases (SULTs), and multidrug resistance-associated proteins (ABCCs) [15,16,17]. Previous studies showed that the genotoxicity of B[a]P was reduced by phase II enzymes conjugated with B[a]P metabolites and that B[a]P was excreted by ABCCs before BPDE-DNA adducts could form [18,19,20]. Nuclear factor erythroid 2-related factor 2 (Nrf2) and pregnane X receptor (PXR) are important transcriptional factors that regulate the expression of anti-genotoxic phase II detoxification enzymes and phase III transporters [18,19,21,22,23]. Generally, Nrf2 protein interacts with kelch-like ECH-associated protein 1 (Keap1) to form a dimer [24]. Exposure to endogenous activators such as reactive oxygen species (ROS) or exogenous agents such as electrophilic xenobiotics induces dissociation of the Nrf2 and Keap1 dimer, resulting in Nrf2 degradation by the proteasome and Nrf2 entry into the nucleus. After translocation into the nucleus, small musculoaponeurotic fibrosarcoma (Maf) proteins and other transcription factors help to activate and stabilize Nrf2, leading to the induction of phase II detoxifying enzymes [25,26].

Silymarin, a natural flavonoid, is a constituent of milk thistle (*Silybum marianum*). It is a metabolic regulator known to have anti-oxidant, anti-inflammatory, anti-cancer, anti-mutagenic, anti-bacterial, and anti-virus effects [27,28,29,30]. Furthermore, silymarin has multiple pharmacological activities and its components include silibinin, silydianine, and silychristin [27]. Recent studies showed that silymarin can reduce B[a]P-induced toxicity by reducing ROS formation in a rat model and can attenuate colorectal, liver, and lung cancer [28,31,32,33]. Another report showed that silymarin also exerts anti-oxidant effects by up-regulating NQO1 and heme oxygenase 1 genes through Nrf2 modulation [33]. However, B[a]P induces DNA damage through BPDE-DNA adduct formation as well as ROS formation, and the mechanism of action through which silymarin reduces the formation of BPDE-DNA adducts has not yet been clearly elucidated. In this study, we show the effect of silymarin on the reduction of genotoxicity through the regulation of B[a]P metabolites and the reduction of BPDE-DNA adduct formation via the modulation of Nrf2 and PXR signaling pathways.

## 2. Results

### 2.1. Attenuation of B[a]P-Induced Cytotoxicity by Silymarin

**Figure 1:**
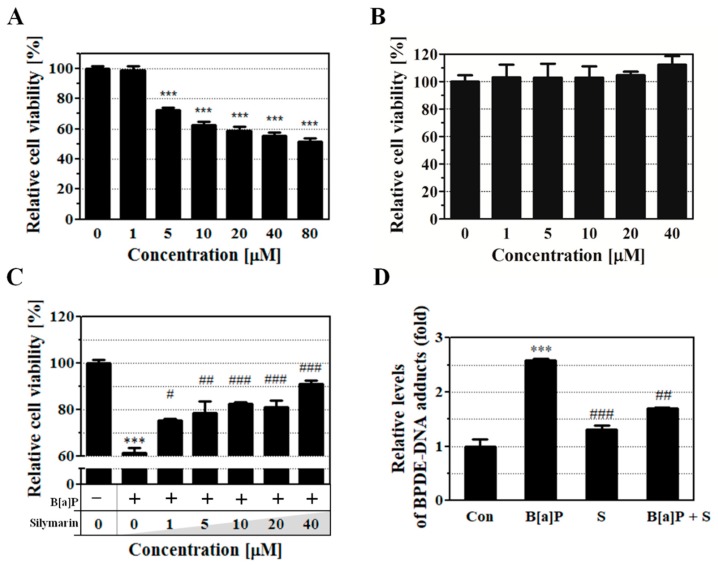
HepG2 cell viability was evaluated by cell viability assay. (**A**,**B**) HepG2 cells were treated with benzo[a]pyrene (B[a]P) or silymarin at various concentrations for 48 h. (**C**) B[a]P-induced cytotoxicity was reduced in cells treated with various concentrations of silymarin for 48 h. (**D**) The inhibitory effect of silymarin on B[a]P-7,8-dihydrodiol-9,10-epoxide (BPDE)-DNA adduct formation was measured by enzyme-linked immunosorbent assay (ELISA). HepG2 cells were treated with B[a]P (10 μM) in the presence or absence of silymarin (40 μM) for 48 h. All treatment group values were significantly different in comparison to the controls (*** *p* < 0.001) and to B[a]P (# *p* < 0.05, ## *p* < 0.01, and ### *p* < 0.001) in Tukey’s multiple comparison test.

Regulation of the dose-dependent production of BPDE in human hepatocytes varies depending on the cell line used for BPDE-DNA adduct research [34]. Therefore, we used the well-characterized HepG2 cell line to study the potential preventive effects of silymarin on BPDE formation, thereby reducing the cell toxicity induced by B[a]P. The toxicity of B[a]P and silymarin on HepG2 cells was evaluated using cell viability assays. B[a]P induced cell death in a dose-dependent manner, whereas silymarin was non-toxic (up to 40 μM for 48 h) compared to no treatment (Figure 1A,B). To evaluate the protective effects of silymarin on B[a]P-induced cytotoxicity, B[a]P was co-applied to HepG2 cells with various concentrations of silymarin. Silymarin restored up to 90% of the cell viability in a dose-dependent manner by reducing B[a]P-induced cytotoxicity. This implies that silymarin has a protective effect against B[a]P-induced cytotoxicity (Figure 1C).

The potential protective effect of silymarin against B[a]P-induced genotoxicity was evaluated. BPDE-DNA adduct formation was measured with a BPDE-DNA adduct enzyme-linked immunosorbent assay (ELISA) kit after treatment with B[a]P (10 μM), silymarin (40 μM), and B[a]P (10 μM) co-administered with silymarin (40 μM). The results showed that B[a]P treatment alone increased the BPDE-DNA adduct level compared to that of the untreated control group. In contrast, B[a]P co-treatment with silymarin significantly decreased the BPDE-DNA adduct level compared to B[a]P treatment alone (Figure 1D). These results suggest that silymarin exerts an anti-genotoxic effect by reducing the formation of BPDE-DNA adducts.

### 2.2. Reduction of Intracellular B[a]P Metabolites by Silymarin

**Figure 2:**
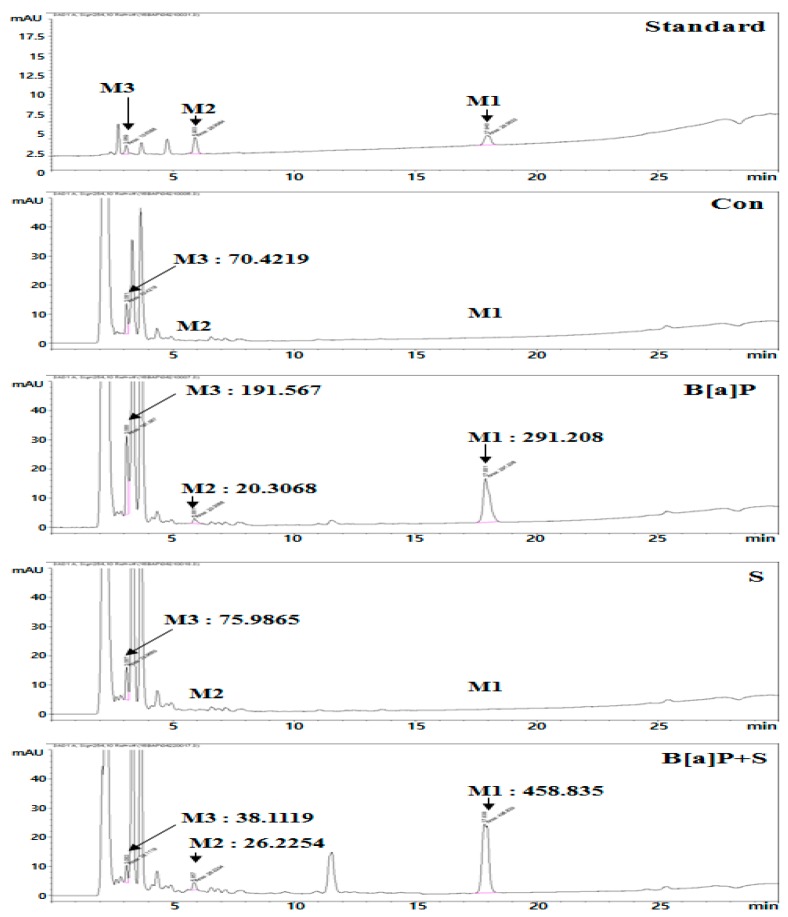
HepG2 cells were incubated with B[a]P (10 μM) and co-treated with silymarin (40 μM) for 48 h. The typical intracellular metabolites of B[a]P were measured by high performance liquid chromatography (HPLC). M1, B[a]P; M2, B[a]P-7,8-dihydrodiol; M3, BPDE.

B[a]P is sequentially metabolized to B[a]P-7,8-dihydrodiol and BPDE; BPDE induces genotoxicity by introducing mutagenic adducts into guanine [12]. The amount of B[a]P and its metabolites B[a]P-7,8-dihydrodiol and BPDE in the treated HepG2 cells were measured using a high performance liquid chromatography (HPLC) system. We found that the calculated amount of BPDE rapidly increased when the cells were treated with B[a]P alone. In contrast, BPDE levels decreased following B[a]P co-treatment with silymarin compared to the levels with B[a]P treatment alone, but the calculated amounts of B[a]P and B[a]P-7,8-dihydrodiol increased (Figure 2). These results suggest that silymarin reduces B[a]P-induced genotoxicity through the excretion of BPDE and inhibition of BPDE-DNA adduct formation.

### 2.3. Modulatory Effect of Silymarin on the Expression of Phase I, II, and III Enzymes

**Figure 3:**
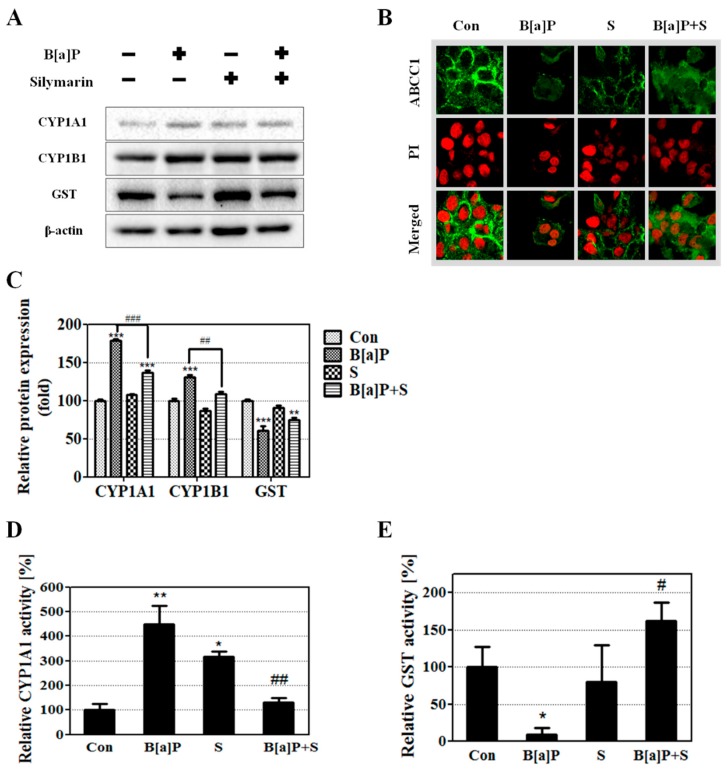
Expression of phase I, II, and III enzymes was induced by incubation for 48 h with B[a]P (10 μM) and co-treatment with silymarin (40 μM). (**A**) Cell lysates were prepared and the level of detoxifying enzymes was measured by Western blot; (**B**) The expression of multidrug resistance-associated protein 1 (ABCC1) was measured using immunocytochemistry. (**C**) Quantitative protein expression was calculated. (**D**,**E**) cellular cytochrome P450 (CYP)1A1 and glutathione S-transferase (GST) were activated and the relative levels of activation were calculated. All values are expressed as mean ± standard error of mean (SEM) (*n* = 3). The relative activation levels of CYP1A1 and GST were significantly different compared to those of the controls (* *p* < 0.05, ** *p* < 0.01, *** *p* < 0.001) and B[a]P (# *p* < 0.05, ## *p* < 0.01, ### *p* < 0.001) in Tukey’s multiple comparison test.

GST enzymes are important for phase II detoxification processes and for the activation of glutathione (GSH) conjugated with BPDE, which is eliminated by ABCCs [18,35]. ABCC1 is involved in the function of efflux pumps, which contribute to GSH-conjugated with xenobiotic (GS-X) excretion. GSH-BPDE conjugates in the cell are eliminated through excretion to the basolateral side by ABCC1 [20,36,37]. In the liver, GSH-BPDE conjugates are transferred to the blood from luminal surfaces through basolateral efflux by ABCC1, and then BPDE is terminally eliminated from the body [37]. The results of B[a]P application showed that B[a]P induced CYP1A1 expression but reduced GST and ABCC1 expression; however, B[a]P co-treatment with silymarin recovered the expression of GST and ABCC1 (Figure 3A–C). The activation of CYP1A1 and GSTs was evaluated using an activity assay kit. Greater activation of GSTs was observed with B[a]P co-administered with silymarin than with B[a]P treatment alone, whereas the activation of CYP1A1 decreased (Figure 3D,E). These data suggest that silymarin reduces B[a]P metabolism and induces BPDE excretion by regulating phase I, II, and III metabolizing enzymes.

### 2.4. Stimulation of Nuclear Translocation of Nrf2 and PXR by Silymarin

**Figure 4:**
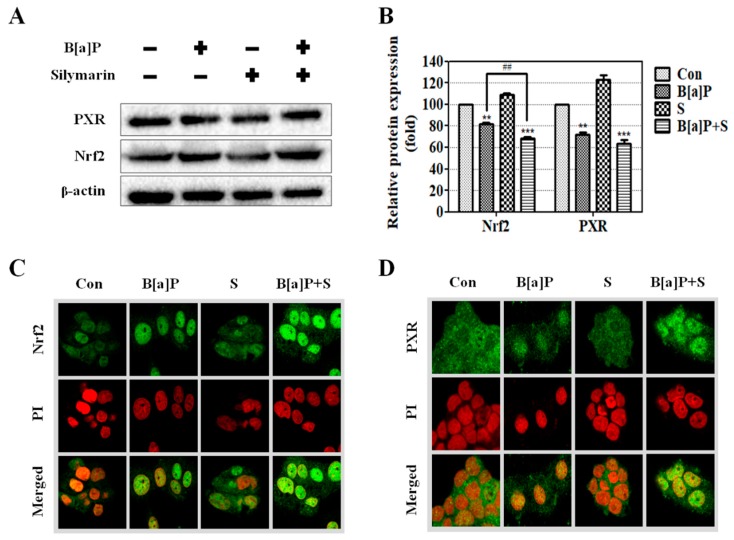
HepG2 cells were treated with B[a]P (10 μM), silymarin (40 μM), and both B[a]P (10 μM) and silymarin (40 μM). (**A**) Nuclear factor erythroid 2-related factor 2 (Nrf2) and pregnane X receptor (PXR) expression was measured using Western blot, and (**B**) the quantitative protein levels were calculated. (**C**,**D**) The translocation levels were measured by immunocytochemistry. DNA was detected by propidium iodide (PI) staining (red).

Nrf2 and PXR are well-known as inducers of phase II detoxification enzyme and phase III transporter expression [17,33,38,39,40]. To elucidate the pathways involved in the silymarin-mediated suppression of BPDE metabolites, the effects of Nrf2 and PXR translocation were evaluated using immunofluorescence staining. The results showed that the protein expression of Nrf2 decreased following B[a]P co-treatment with silymarin, whereas PXR expression was not significantly altered compared to the levels observed with B[a]P treatment alone (Figure 4A,B) However, B[a]P co-treatment with silymarin significantly increased the translocation of Nrf2 and PXR compared to B[a]P alone (Figure 4C,D). These results indicated that silymarin induces the translocation of Nrf2 and PXR, which are mainly involved in pathways related to the elimination of BPDE metabolites.

### 2.5. Reduction of BPDE-DNA Adduct by Silymarin is Dependent on Nrf2

**Figure 5:**
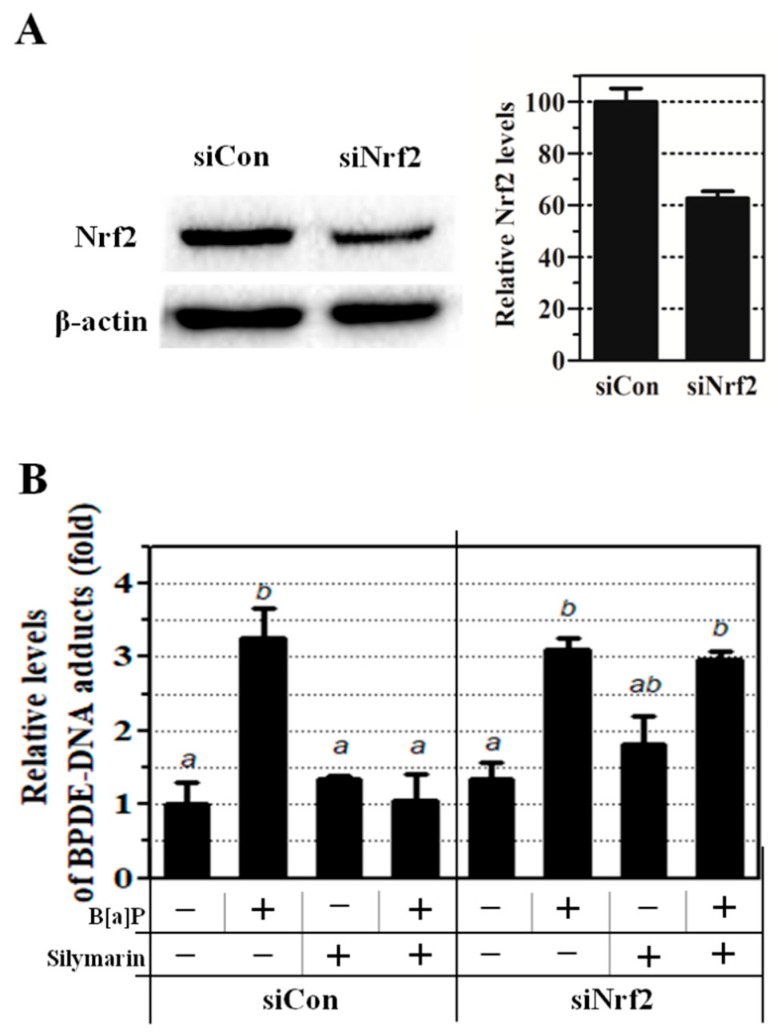
Knockdown of HepG2 cells was performed using Nrf2-small interfering RNA (siRNA) (siNrf2) and control siRNA (siCon). (**A**) Knockdown of cells was confirmed by Western blot. (**B**) Transfected HepG2 cells were exposed to B[a]P for 48 h. BPDE-DNA adduct formation was measured by enzyme-linked immunosorbent assay (ELISA). Each letter indicates significantly (*p* < 0.05) different values (a, b, ab—mean values with the same letters for each level are not significantly different).

Previous results showed that silymarin reduces BPDE-DNA adduct formation through the Nrf2 signaling pathway. We confirmed that the preventive effect of Nrf2 was inhibited by knockdown of Nrf2 expression using specific small interfering RNA (siRNA). Depletion of Nrf2 was confirmed by Western blot (Figure 5A). Co-treatment of HepG2 cells with B[a]P and silymarin resulted in the reduction of BPDE-DNA adduct formation with control siRNA (siCon). However, this effect of silymarin on BPDE-DNA adduct formation was eliminated by Nrf2 knockdown (Figure 5B). These results confirmed that BPDE-DNA adduct formation was mainly reduced by silymarin via the up-regulation of Nrf2 translocation.

## 3. Discussion

B[a]P is a group I carcinogen that is widely produced in cigarette smoke, charbroiled food, and other sources. It activates several types of cancer, mainly by causing DNA damage through BPDE-DNA adduct and ROS formation [41,42]. Unfortunately, we are constantly exposed to low-doses of B[a]P, which induces cancer in the body through its conversion to the BPDE metabolite [43,44]. Research on the protective effects of natural compounds against B[a]P-induced damage is currently ongoing to identify methods to prevent cancer [45,46,47,48,49].

Silymarin, a natural compound derived from milk thistle (*Silybum marianum*) is widely used in health supplements, medicines, and medical supplies related to liver disease and cancer, and has no reported side-effects [50,51,52]. Previous studies on silymarin focused on its role as an anti-oxidant and its anti-inflammatory properties against B[a]P-induced ROS formation via modulating detoxifying enzymes in vivo [28]. However, B[a]P-induced genotoxicity is mainly caused by damage from BPDE-DNA adducts as well as ROS-based DNA damage. Additionally, the metabolic counteractive effects of silymarin on B[a]P-induced toxicity have not yet been studied. In this study, we provide evidence that silymarin reduces B[a]P-induced genotoxicity by modulating phase detoxification enzymes and eliminating B[a]P metabolites via the Nrf2 and PXR signaling pathways. Our results show that co-application of B[a]P with silymarin recovers the cell viability levels (Figure 1C). Previous reports showed that silymarin possesses anti-oxidant effects and attenuates B[a]P-induced ROS damage [27,53]. However, we focused on the attenuation of BPDE-DNA adduct formation by silymarin in this study. Our results provide new insight that novel mechanisms of silymarin modulate the attenuation of BPDE, facilitating anti-genotoxic effects against B[a]P. BPDE is the ultimate metabolite of B[a]P and is produced through genotoxic interactions with DNA to induce BPDE-DNA adduct formation. The present study confirms that silymarin inhibits BPDE-DNA adduct formation compared to the effects of B[a]P alone (Figure 1D). This result indicates that silymarin attenuates B[a]P-induced genotoxicity by inhibiting BPDE-DNA adduct formation.

To confirm the mechanism of BPDE-DNA adduct formation by silymarin, we evaluated the amount of B[a]P metabolites produced. Silymarin co-treatment with B[a]P resulted in greater production of B[a]P and B[a]P-7,8-dihydrodiol than B[a]P alone. However, the amount of BPDE was markedly decreased (Figure 2), consistent with the formation of fewer BPDE-DNA adducts as the amount of BPDE decreased (Figure 1D). Additionally, the results suggest that silymarin reduces B[a]P metabolism by inhibiting the transition of B[a]P to BPDE. A previous report indicated that direct exposure of cells to BPDE resulted in instant formation of DNA adducts, whereas B[a]P-exposed cells required multiple enzymatic steps for B[a]P conversion to BPDE [54]. This result indicates that the inhibition of BPDE formation is important to prevent B[a]P-induced genotoxicity by modulating the necessary enzymatic steps. Therefore, we hypothesized that the inhibition of B[a]P conversion to BPDE will be regulated by enzymatic steps.

CYP1A1 is considered as playing a key role in B[a]P metabolism involving both BPDE formation and reduction [55]. Further, abnormal activation of phase I enzymes may have an adverse effect by inducing toxicity and causing cancer in the body [56]. Our results showed that co-treatment with silymarin reduced CYP1A1 and CYP1B1 protein level compared to B[a]P treatment alone (Figure 3A,C). Moreover, silymarin reduced CYP1A1 activity to levels similar to those of the control group (Figure 3D). These results indicated that CYP1A1 and 1B1 contribute to the inhibition of both B[a]P metabolism and BPDE generation by silymarin.

Xenobiotics such as B[a]P are mainly converted to water-soluble metabolites that are easily eliminated from the body [57]. These defense systems are modulated by phase detoxification enzymes such as GSTs, SULTs, uridine 5′-diphospho-glucuronosyltransferases (UGTs), and ABCCs that induce the excretion of toxicants through urine [58]. Previous studies showed that B[a]P-induced genotoxicity was reduced by the modulation of phase detoxifying enzymes before BPDE-DNA adduct formation [17,47]. GSTs are typical phase II detoxifying enzymes and are important in reducing B[a]P-induced DNA damage by inhibiting the formation of DNA adducts and 8-oxo-G [59]. Additionally, GSTs are known to promote GSH conjugation with BPDE and inhibit BPDE-DNA adduct formation [60]. Our results showed that the expression and activity of GSTs were promoted by silymarin compared to the observations with B[a]P alone (Figure 3A,C,E). Reduced BPDE production is caused by two factors: (1) inhibition of B[a]P conversion to BPDE by the regulation of CYP1A1 and CYP1B1, and (2) elimination of BPDE metabolites via the induction of BPDE conjugation with GSH through the modulation of GST.

GSH conjugated with metabolites enhances the water solubility of B[a]P metabolites, facilitating the excretion of BPDE by ABCCs [61]. Previous studies showed that BPDE was detoxified when conjugated with GSH and excreted by ABCCs [20,62]. Specifically, ABCC1 and ABCC2 are mainly involved in the excretion of GSH-BPDE conjugates, with ABCC2 mediating the apical excretion and ABCC1 mediating the basolateral excretion [20]. We confirmed that ABCC1 mRNA expression, but not ABCC2 mRNA expression, is regulated by silymarin based on microarray data (data not shown). Another study also showed that the prevention of xenobiotic toxicity by ABCC1 is important in several types of tissues and resulted in the direct excretion of GS-X [63,64]. Our results demonstrated that the level of ABCC1 is reduced by B[a]P treatment and restored by co-treatment with silymarin (Figure 3B). These results indicate that the mechanism underlying the reduction of B[a]P-induced genotoxicity involves the activation of GST to enhance GSH conjugation with BPDE, excretion of BPDE into the blood from the liver by ABCC1, and elimination from the body through urine. As a consequence, silymarin induces the up-regulation of GST and ABCC1, facilitating BPDE conjugation with GSH and excretion of the conjugate, and inhibits the conversion of BPDE from B[a]P by regulating CYP1A1 and CYP1B1. Therefore, these results confirm that silymarin reduces B[a]P-induced genotoxicity through the inhibition of BPDE-DNA adduct formation.

Aryl hydrocarbon receptor (AhR), one of major transcriptional factor of phase I enzymes, stimulates expression of CYP enzymes by binding with xenobiotic response element (XRE) [65,66] and induces to B[a]P metabolism. We confirmed that B[a]P transition to BPDE was decreased (Figure 2) whereas CYP1A1 and 1B1 mRNA level were increased by silymarin. Our results indicate that the anti-genotoxic effect of silymarin is due to the activity of CYP enzymes, not mRNA expression level. Nrf2 and PXR are major transcriptional factors of phase II detoxification enzymes and phase III transporters, and they are reported to modulate GSTs, NQO1, UGTs, and ABCCs [67,68,69]. A previous study demonstrated that Nrf2 plays a critical role in drug metabolism by regulating phase I, II, and III enzymes in the liver [70]. In addition to this, Nrf2 is important for maintaining GSH and regulating the expression of phase I, II, and III enzymes, thereby facilitating the elimination of xenobiotics [71]. Moreover, nuclear translocation of Nrf2 interactions with antioxidant response element (ARE) induces phase detoxifying enzymes [71]. Our results show that the expression of Nrf2 by B[a]P is slightly decreased by co-exposure with silymarin, whereas nuclear translocation of Nrf2 is increased significantly (Figure 4A–C). Accordingly, our results suggest that silymarin induces phase detoxifying enzymes via Nrf2. A previous study showed that PXR regulated the detoxifying enzymes against B[a]P and reduced toxicity in the liver [22]. This report indicates that PXR is also related to the anti-genotoxic effect of silymarin. Therefore, these results suggest that increased Nrf2 and PXR translocation regulates phase detoxifying enzymes such as GSTs and ABCC1, which then facilitate the elimination of B[a]P metabolites.

**Figure 6:**
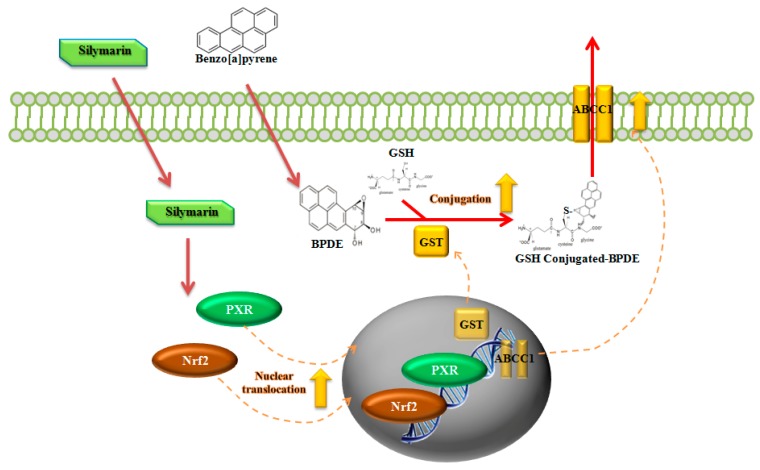
Schematic of reduction of B[a]P-induced genotoxicity by silymarin through Nrf2 and PXR. Silymarin induces Nrf2 and PXR translocation and can regulate the expression of GSTs and ABCC1, enhancing GSH conjugation with BPDE to facilitate the excretion process.

A previous study showed that the inhibition of Nrf2 increased B[a]P toxicity and decreased the expression of several GSTs [72]. To confirm that silymarin regulates the Nrf2 signaling pathway, which is related to the attenuation of BPDE-DNA adduct formation, we demonstrated that knockdown of Nrf2 expression by RNA interference abolishes the inhibition of BPDE-DNA adduct formation by silymarin (Figure 5B). This indicates that the induction of Nrf2 translocation by silymarin plays a key regulatory role against B[a]P-induced genotoxicity. Previous studies supported that Nrf2 was closely related to the induction of GSTs and ABCC1 through the regulation of Nrf2 expression [73,74]. These reports support our findings that silymarin modulates Nrf2, which causes B[a]P detoxification through the regulation of phase II and III enzymes. These results confirm that silymarin exerts anti-genotoxic effects against B[a]P-induced toxicity through the up-regulation of Nrf2 and PXR translocation (Figure 6).

Previous reports indicated that silymarin attenuated DNA damage by reducing ROS damage. However, BPDE also causes DNA damage by forming BPDE-DNA adducts, which induce toxic and pathologic cellular changes. Our results clearly show that the signaling pathways inhibit B[a]P conversion to BPDE by modulating phase I enzymes and accelerate BPDE conjugation with GSH by regulating phase detoxification enzymes that counteract B[a]P metabolites. In this manner, silymarin can reduce B[a]P-induced genotoxicity by reducing DNA damage through the inhibition of BPDE interactions with DNA.

## 4. Methods

### 4.1. Chemicals and Reagents

Silymarin, B[a]P, dimethyl sulfoxide (DMSO), 4,6-diamidino-2-phenylindole dihydrochloride (DAPI), and Triton X-100 were purchased from Sigma-Aldrich Chemical (St. Louis, MO, USA). Minimum essential medium (MEM), fetal bovine serum (FBS), penicillin/streptomycin, trypsin-ethylenediaminetetraacetic acid (EDTA), and sodium pyruvate were purchased from Welgene (Daegu, Korea). Phosphate-buffered saline was purchased from Biosesang (Seongnam, Korea). Fluorescent mounting medium was purchased from Dako (Carpinteria, CA, USA). Antibodies (anti-Nrf2, PXR, GST, CYP1A1, ABCC1, and β-actin), horseradish peroxidase-conjugated anti-rabbit immunoglobulin G (IgG) and siRNA transfection reagent, siRNA transfection medium, control siRNA, and Nrf2-siRNA were purchased from Santa Cruz Biotechnology (Santa Cruz, CA, USA). Alexa 488-conjugated anti-rabbit secondary antibody was purchased from Cell Signaling Technology (Beverly, MA, USA).

### 4.2. Cell Culture and Treatment

The HepG2 cell line was purchased from the American Type Culture Collection (Manassas, VA, USA). HepG2 cells were cultured in a 100 mm^2^ cell culture dish with MEM containing 10% FBS, 100 U/mL penicillin, 100 μg/mL streptomycin, and 1 mM sodium pyruvate at 37°C in a humidified atmosphere of 5% CO_2_. To determine the effects of the treatment conditions, the cells were incubated with various concentrations of B[a]P and silymarin in MEM for 48 h. The cells were subjected to further analysis or harvested to prepare cell-free extracts.

### 4.3. Cell Viability Assay

We performed cell viability assays to evaluate the general cytotoxicity of B[a]P and silymarin on HepG2 cells. HepG2 cells with a density of 1 x 10^4^ cells/well with MEM were seeded in 96-well plates with B[a]P (0, 1, 5, 10, 20, 40 μM) or silymarin (0, 1, 5, 10, 20, 40 μM) for 48 h. EZ-CYTOX reagent (DOGEN, Daejeon, Korea) was added to each well and the cells were incubated for 2 h. Absorbance measurement at 450 nm was carried out by using a microplate reader (Molecular Devices, San Jose, CA, USA) and the cell viability levels of the B[a]P and silymarin treatment groups were evaluated compared to those of the non-treatment groups.

### 4.4. BPDE-DNA Adduct Formation Analysis

HepG2 cells were seeded with MEM in 6-well plates and were treated with 10 μM B[a]P in the presence or absence of 40 μM silymarin for 48 h. DNA was extracted from the HepG2 cells at the end of the treatment period using the QIAamp DNA Mini Kit (Qiagen, Stanford, CA, USA) according to the manufacturer’s instructions. The isolated DNA was analyzed for BPDE-DNA adduct formation using a BPDE-DNA adduct ELISA kit (Cell Biolabs, San Diego, CA, USA) according to the manufacturer’s instructions. The relative BPDE-DNA adduct levels were measured using a microplate reader with absorbance at 450 nm.

### 4.5. Metabolite Extraction and HPLC Analysis Conditions

To extract metabolites, cells treated with B[a]P and/or silymarin in 100 mm^2^ cell culture dishes for 48 h were dissolved with ethyl acetate and homogenized. The dissolved cells were evaporated using a vacuum centrifuge and re-dissolved with 50% acetonitrile/0.1% acetic acid. After this, 30 μL was injected into an Agilent HPLC Hewlett Packard 1100 series (Hewlett-Packard, Palo Alto, CA, USA). Chromatography was performed using a Kinetex C18 column (4.6 mm X 250 mm, 5 μm, Phenomenex, Torrance, CA, USA) at 25 °C with a flow rate of 1.0 mL/min. HPLC separation was performed using the following linear gradient: 25 to 30 min for 100% acetonitrile in 0.1% acetic acid, and 0 to 40 min for 50% acetonitrile in 0.1% acetic acid (with solvent A, 0.1% acetic acid in distilled water and solvent B, 0.1% acetic acid/acetonitrile in distilled water). For each sample, the retention time and fragmentation patterns were obtained from reference standards of B[a]P, B[a]P-7,8,dihydrodiol, and BPDE, which were purchased from the MRIGlobal Chemical Carcinogen Repository (Kansas City, MO, USA).

### 4.6. CYP1A1 Activity Assay

HepG2 cells were seeded at a density of 1 × 10^4^ cells into each well of 96-well plates with MEM and were treated with 10 μM B[a]P in the absence or presence of 40 μM silymarin for 48 h. CYP1A1 activity was measured using a CYP1A1 activity assay kit (Promega, Madison, WI, USA) according to the manufacturer’s instructions. The luminescence level of CYP1A1 was measured using an Infinite 200 PRO multi-well plate reader (Tecan, Mannedorf, Switzerland).

### 4.7. GST Activity Assay

GST activity was measured using a GST activity assay kit (Cayman, Ann Arbor, MI, USA) according to the manufacturer’s instructions. HepG2 cells were seeded with MEM in 96-well plates at a density of 1 x 10^4^ cells in each well and were treated with 10 μM B[a]P in the absence or presence of 40 μM silymarin for 48 h. The relative activation of GST was measured by using an Infinite 200 PRO multi-well plate reader (Tecan, Mannedorf, Switzerland).

### 4.8. Immunofluorescence Staining

HepG2 cells were seeded on coverslips in a 6-well cell culture plate. The cells were fixed with 4% formaldehyde for 15 min and treated with 0.25% Triton X-100 after the formaldehyde was discarded. The cells were sequentially incubated with primary antibody for 1 h and Alexa 488-conjugated anti-rat secondary antibody for 1 h. The cells with attached antibody were stained with DAPI. After DAPI staining, fluorescence mounting medium was applied to a glass slide on which was placed a coverslip with the attached cells. The fluorescence images were obtained by confocal microscopy (Olympus, Tokyo, Japan) and quantitative analysis was performed using ImageJ software (Bethesda, MD, USA).

### 4.9. Western Blot Analysis

Total cell lysates were prepared in radioimmunoprecipitation assay (RIPA) buffer (50 mM Tris-HCl pH 7.4, 150 mM NaCl, 1% Nonidet P-40, 0.25% sodium deoxycholate) containing protease inhibitor cocktail, phosphatase inhibitor cocktail 2, and phosphatase inhibitor cocktail 3 (Sigma-Aldrich, St. Louis, MO, USA). Total cell proteins (30 µg) were separated by 10% sodium dodecyl sulfate-polyacrylamide gel electrophoresis, transferred to a polyvinylidene difluoride membrane (Millipore, Billerica, MA, USA), and hybridized with their respective primary antibodies. The membrane was incubated with secondary antibodies and the immunoreactive proteins bound to the antibodies were detected with enhanced chemiluminescent (ECL) Plus Western blotting detection reagents (Amersham Bioscience, Buckinghamshire, United Kingdom). Images were acquired using a Bio-Rad ChemiDoc XRS (Hercules, CA, USA) and quantified by Quantity One Image Software (Hercules, CA, USA).

### 4.10. Statistical Analysis

Each experiment was repeated at least three times. All data were expressed as means ± standard error of the mean (SEM). Significant differences among groups were determined by using one-way ANOVA with Tukey’s multiple comparison test. Statistical significance was considered as *p* < 0.05.

## 5. Conclusions

In summary, our results show that the modulation of B[a]P detoxification reduces intracellular B[a]P metabolites, and thereby prevents the formation of BPDE-DNA adducts. Silymarin reduces BPDE by attenuating B[a]P conversion to BPDE and accelerates BPDE detoxification and elimination by modulating phase I, II, and III enzymes via Nrf2 and PXR pathways. In particular, knockdown of the Nrf2 gene abolishes the attenuation of BPDE-DNA adduct formation by silymarin. We confirmed that the Nrf2 signaling pathway is mainly related to the inhibition of BPDE-DNA adduct formation by silymarin. These results suggest that silymarin has anti-genotoxicity properties against B[a]P through the inhibition of BPDE-DNA adduct formation.
